# Autism Spectrum Disorder (ASD) with and without Mental Regression Is Associated with Changes in the Fecal Microbiota

**DOI:** 10.3390/nu11020337

**Published:** 2019-02-05

**Authors:** Julio Plaza-Díaz, Antonio Gómez-Fernández, Natalia Chueca, María José de la Torre-Aguilar, Ángel Gil, Juan Luis Perez-Navero, Katherine Flores-Rojas, Pilar Martín-Borreguero, Patricio Solis-Urra, Francisco Javier Ruiz-Ojeda, Federico Garcia, Mercedes Gil-Campos

**Affiliations:** 1 Department of Biochemistry and Molecular Biology II, School of Pharmacy, University of Granada, 18071 Granada, Spain; jrplaza@ugr.es (J.P.-D.); agil@ugr.es (Á.G.); 2 Institute of Nutrition and Food Technology “José Mataix”, Center of Biomedical Research, University of Granada, 18016 Armilla, Granada, Spain; 3 Instituto de Investigación Biosanitaria IBS.GRANADA, Complejo Hospitalario Universitario de Granada, 18014 Granada, Spain; naisses@yahoo.es (N.C.); fegarcia@ugr.es (F.G.); 4 Pediatric Research and Metabolism Unit, Reina Sofia University Hospital, Maimónides Institute for Biomedical Research of Córdoba (IMIBIC), University of Córdoba, 14010 Córdoba, Spain; antoniogofedez@hotmail.com (A.G.-F.); delatorremj4@gmail.com (M.J.d.l.T.-A.); katherine1.flores@gmail.com (K.F.-R.); mercedes_gil_campos@yahoo.es (M.G.-C.); 5 CIBEROBN (CIBER Physiopathology of Obesity and Nutrition), Instituto de Salud Carlos III, 28029 Madrid, Spain; 6 Department of Child and Adolescent Clinical Psychiatry and Psychology, Reina Sofia University Hospital, Maimónides Institute for Biomedical Research of Córdoba (IMIBIC), 14010 Cordoba, Spain; pmartin.psicologa@gmail.com; 7 PROFITH “PROmoting FITness and Health through physical activity” research group, Department of Physical Education and Sport, Faculty of Sport Sciences, University of Granada, 18071 Granada, Spain; patricio.solis.u@gmail.com; 8 IRyS Research Group, School of Physical Education, Pontificia Universidad Católica de Valparaíso, Valparaiso 2374631, Chile; 9 RG Adipocytes and metabolism, Institute for Diabetes and Obesity, Helmholtz Diabetes Center at Helmholtz Center Munich, 85748 Garching, Munich, Germany; francisco.ruiz@helmholtz-muenchen.de

**Keywords:** autism spectrum disorder, children, intestinal microbiota, nutrients

## Abstract

New microbiome sequencing technologies provide novel information about the potential interactions among intestinal microorganisms and the host in some neuropathologies as autism spectrum disorders (ASD). The microbiota–gut–brain axis is an emerging aspect in the generation of autistic behaviors; evidence from animal models suggests that intestinal microbial shifts may produce changes fitting the clinical picture of autism. The aim of the present study was to evaluate the fecal metagenomic profiles in children with ASD and compare them with healthy participants. This comparison allows us to ascertain how mental regression (an important variable in ASD) could influence the intestinal microbiota profile. For this reason, a subclassification in children with ASD by mental regression (AMR) and no mental regression (ANMR) phenotype was performed. The present report was a descriptive observational study. Forty-eight children aged 2–6 years with ASD were included: 30 with ANMR and 18 with AMR. In addition, a control group of 57 normally developing children was selected and matched to the ASD group by sex and age. Fecal samples were analyzed with a metagenomic approach using a next-generation sequencing platform. Several differences between children with ASD, compared with the healthy group, were detected. Namely, *Actinobacteria* and *Proteobacteria* at phylum level, as well as, *Actinobacteria*, *Bacilli*, *Erysipelotrichi*, and *Gammaproteobacteria* at class level were found at higher proportions in children with ASD. Additionally, *Proteobacteria* levels showed to be augmented exclusively in AMR children. Preliminary results, using a principal component analysis, showed differential patterns in children with ASD, ANMR and AMR, compared to healthy group, both for intestinal microbiota and food patterns. In this study, we report, higher levels of *Actinobacteria*, *Proteobacteria* and *Bacilli*, aside from *Erysipelotrichi*, and *Gammaproteobacteria* in children with ASD compared to healthy group. Furthermore, AMR children exhibited higher levels of *Proteobacteria*. Further analysis using these preliminary results and mixing metagenomic and other “omic” technologies are needed in larger cohorts of children with ASD to confirm these intestinal microbiota changes.

## 1. Introduction

The advent of new sequencing technologies has stimulated the beginning of new research to ascertain the connections between the microbial communities that reside in our gut and some physiological and pathological conditions. The microbiota, defined as the full collection of microbes (bacteria, fungi, viruses, among others) that naturally exist within a particular biological niche, is estimated to contain 500–1000 species [1,2,3], and has an important impact on human health. In particular, the gut microbiota may play a key role in many essential processes in health and disease via the activity of the gut-brain axis, possibly contributing to autism spectrum disorders (ASD), Alzheimer’s disease, Parkinson’s disease, depression, and anxiety disorders, among others [4].

ASD is a severe neurodevelopmental disorder that impairs child’s ability to communicate and interact with others. Children with neurodevelopmental disorders, including ASD, are regularly affected by gastrointestinal problems and dysbiosis of the gut microbiota [5]. Autism diagnoses have increased rapidly over the last decade (currently 1 in 59 births, versus 1 in 150 reported in 2000 in the United States [6]). In the case of Spain, the estimated prevalence is 1.55% in preschoolers and 1.00% in school-age children, and male-to-female ratio was around 4:1 [7].

The pathophysiology of ASD still remains largely unknown. However, a disorder of the microbiota–gut–brain axis is emerging as a prominent factor in the generation of autistic behaviors [8]. Evidence so far suggests prognosis is not pre-determined and there is a dynamic component of disease. Many studies highlight the possibility for environmental risk factors and associated medical co-morbidities to contribute to core neurobehavioral symptoms of the disorder [9,10,11]. Co-morbid gastrointestinal symptoms in some subsets of ASD individuals include diarrhea/constipation, abdominal pain and gastric reflux. In relation with this phenotype, deficient integrity of the gut epithelium and increased intestinal permeability have been reported [12]. In addition, evidence from animal models suggests that certain microbial shifts in the gut may produce changes consistent with the clinical picture of autism, with proposed mechanisms including toxin production, aberrations in fermentation processes/products, and immunological and metabolic abnormalities [13].

Currently, animal model evidence [14,15], as well as more limited human studies have demonstrated that signaling along the gut-microbiome-brain axis is a critical regulator of both central nervous system and immune function [16,17]. Previous studies have shown microbial changes in the intestinal microbiota of children with ASD compared with healthy subjects in certain bacteria genera belonging to the phyla *Bacteroidetes*, such as *Bacteroides*, *Barnesiella*, *Odoribacter*, *Parabacteroides*, *Prevotella* and *Alistipes*, and belonging to *Proteobacteria* (e.g., *Proteus*, *Parasutterella*). In contrast, *Bifidobacterium* species, belonging to the phylum *Actinobacteria*, were decreased [18,19]. *Akkermansia muciniphila* (phylum *Verrucomicrobia*), was found in high levels in children suffering ASD [20,21]. However, these studies have reported a low number of evaluated samples (from 11 to 33 children), and none of the aforementioned defined appropriately the diversity of phenotypes within the ASD children population. It is well established that children with ASD do not constitute a homogeneous clinical group, and many different pathologies show a similar constellation of behavioral symptoms that converge within ASD [22] for example, the existence of developmental regression in some children with ASD has been corroborated by multiple studies [23]. Many studies report dysbiosis of the gut microbiota in ASD individuals [24,25]. Alterations in the gut microbiota are observed in ASD individuals compared to neurotypical controls. Increased clostridia species in autism have been reported in several studies and microbiome alterations that might contribute to the development of autism include altered immune function and bacterial metabolites [26]. Other authors have hypothesized that an increase in *Candida* spp. could be looked at as precocious index of intestinal dysbiosis. However, none correlation was observed between detected counts of *Candida* spp. and gastrointestinal symptoms in ASD children [24]. ASD severity was also linked to a reduction in short-chain fatty acids (SCFAs), including acetate, propionate and butyrate [27], which are modulated by gut microbes. In the case of mental regression, the information is scarce.

The gastrointestinal tract is an organ that is co-dominated by the central nervous system (CNS), the autonomic nervous system, and the enteric nervous system (ENS). Regulation of enteric nerves consists of different levels of nervous regulation [28]. This type of neuroendocrine network, which connects the gastrointestinal tract with the CNS at different levels, is the structural basis for the function of the microbiota gut-brain axis. Disorders of neurological control, such as ASD, at any level will affect the function of the gut and brain. The gut has a direct neural connection with the brain through the vagus nerve, and bacteria can stimulate the afferent neurons of the ENS [29]. The vagus signal from the gut can trigger an anti-inflammatory response against the sepsis induced by microorganisms. Gut microorganisms can affect brain functions through the vagus nerve; after a vagotomy, the microorganisms will not be able to regulate behaviors [30,31].

Glial cells (in particular microglia and astrocytes) influence synapse formation and function [32,33]. Microglia are immune cells in the CNS, and studies have found that the metabolism of gut microorganisms can regulate the maturation and function of microglia, thereby affecting CNS function [34]. Both elevated microglial activation and altered microglia to neuron spatial distribution patterns have been observed in the cerebral cortex and cerebellum of postmortem ASD brains [35,36] and surrogate markers of increased microglial activation have shown by positron-emission tomography imaging of living ASD individuals [37].

Interestingly, Erny and colleagues have demonstrated that the microbiome is required for proper development and function of adult brain microglia [34]. Microglia from germ-free mice exhibit altered morphology, with longer processes and increased branching, and altered transcriptomes including down-regulation of cell activation genes, reduction of genes for type 1 IFN receptor signaling, and up-regulation of microglia transcription and survival factors compared to those isolated from conventionally-colonized controls. Re-colonization of adult gnotobiotic mice with a conventional gut microbiota or supplementation with SCFAs corrects these deficiencies in microglial activation [34,38]. Recently, Lu et al. have reported that growth-associated microbiota can influence early neuron and oligodendrocyte development and this effect may be mediated by effects on neuroinflammation and circulating IGF-1 [39].

In addition, diet might contribute to the development of phenotypic diversity in ASD children influencing their behavior [40]. Recently, changes in brain structure were found to be associated with diet-dependent changes in gut microbiome populations using a machine learning classifier for quantitative assessment of the strength of microbiome-brain region associations [41].

Scarce information about children with ASD intestinal microbiota changes is available. Furthermore, there is a lack of knowledge about how mental regression would affect eating behavior and intestinal microbiota in ASD children. Thus, the aim of the present study was to evaluate the fecal metagenomic profiles in children with ASD and compare them with healthy participants, in order to ascertain how other important variable in ASD as mental regression could influence the intestinal microbiota profile. For this reason, a sub-classification in children with ASD by mental regression (AMR) and no regression (ANMR) phenotype was performed to augment the knowledge of gut-brain-microbiota axis, highlighting the potential impact of diet and metagenomic effects in neuropathologies.

## 2. Material and Methods

### 2.1. Ethical Statement

The present study was a cross-sectional case-control study and was approved by the Clinical Research and Bioethics Committee at Reina Sofia University Hospital respecting the fundamental principles established in the Declaration of Helsinki. The selected subjects were incorporated into the study after all inclusion criteria were fulfilled and informed written consents from the children’s legal guardians were obtained.

### 2.2. Participants

Fifty-seven children with ASD were initially selected for this descriptive observational study (the recruitment started during 2015 in the Department of Psychology of the Pediatric Service). Of those, three children were excluded for not meeting the diagnostic criteria for ASD during the subsequent follow-up interviews at 18 months [22]. Such interviews (Autism Diagnostic Interview—Revised ADI-R) were a follow-up to verify if the individuals continued meeting the Diagnostic and Statistical Manual of Mental Disorders (DSM-5) criteria.

All selected children with ASD were between 2–6 years old with an agreed clinical diagnosis using the criteria of the International Classification Disease 10th Edition for ASD [42] and DSM-5 [43]. The diagnosis was confirmed by scores above the cut-off points of two tests, the Autism Diagnostic Observation Schedule test, with revised algorithms, and the Pervasive Developmental Disorders (Behavior Inventory [PDDBI]), the latter as a means of obtaining a measure of the severity of ASD. The ASD group was also classified according to whether the children presented developmental delay (a score lower than 70 in the cognitive quotient of the Battelle developmental test) or not. Within the ASD group, 20 children were classified as AMR and 32 as ANMR: two children could not be clearly classified into either subgroup. The subdivision (ANMR and AMR) was based on the presence or absence of developmental regression during the first two years of life, which was assessed by a five-item questionnaire following the guide used by the ADI-R clinical interview for the evaluation of this process [44]. Children with ASD who obtained a score equal to three or greater, were included in the mental regression group, whereas those with a score of less than this value were included in the non-mental regression group [22].

Children with ASD presenting other known neurological, metabolic or genetic diagnoses were excluded, as were children with medical treatment for autism-related behavioral comorbidities that may interfere with the results, such as sedatives, muscle relaxants or similar.

Additionally, a control group of 57 normally developing children was selected and matched to the ASD group by sex and age. Children in the control group were chosen from those who came to the hospital for pre-anesthesia for minor surgery (mainly hernias). The clinical and analytical absence of illness in this group of healthy children was confirmed.

Finally, the metagenomic fecal analysis included 18 children classified as AMR, 30 classified as ANMR and 57 as control group.

### 2.3. Assessment of Diet

Guardians completed a 24-h dietary record during the intervention period. In addition, one of the authors (KF) applied a validated semiquantitative food frequency questionnaire. Energy consumption and dietary intakes of macro- and micronutrients data were estimated using the Odimet software (University of Santiago de Compostela, Spain; http://www.odimet.es/). The Spanish Community Nutrition Society (SENC) Guidelines for scholar age were used to compare the actual food intake of ASD and healthy children with the standard recommendations [45].

### 2.4. Metagenomic Analysis

#### 2.4.1. DNA Extraction

Fecal samples were collected in plastic sterile containers from each volunteer and then transferred to −80 °C until analysis. Fecal samples were homogenized in a Stomacher-400 blender. DNA was extracted using a QIAamp DNA Stool Mini Kit (QIAGEN, Barcelona, Spain) as directed by the manufacturer, with the exception that samples were mixed with the lysis buffer and incubated at a temperature of 95 °C instead of 70 °C to ensure lysis of both Gram-positive and Gram-negative bacteria. Quantification was conducted with a NanoDrop ND-1000 spectrophotometer (Thermo Fisher Scientific, DE, USA). The, DNA yield was determined by measuring absorbance ratios spectrophotometrically, and the measurement includes A260/280 nm for protein contamination and A260/230 nm for salt and phenol contamination.

#### 2.4.2. Sequencing Analysis

The extracted DNA was PCR amplified using the primer pairs, 16S Amplicon PCR Forward Primer: 5′TCGTCGGCAGCGTCAGATGTGTATAAGAGACAGCCTACGGGNGGCWGCAG, and 16S Amplicon PCR Reverse Primer: 5′GTCTCGTGGGCTCGGAGATGTGTATAAGAGACAGGACTACHVGGGTATCTAATCC targeting the V3 and V4 hypervariable regions of the bacterial 16S rRNA gene [46]. All PCRs were performed in 25 μL reaction volumes containing 12.5 μL 2X KAPA HiFi Hotstart ready mix (KAPA Biosystems, Woburn, MA, USA), 5 μL of each forward and reverse primers (1 μM) and 2.5 μL of extracted DNA (10 ng) under the following cycling conditions: initial denaturation at 95 °C for 3 min, followed by cycles of denaturation at 95 °C for 30 s, annealing at 55 °C for 30 s, and elongation at 72 °C for 30 s, with a final extension at 72 °C for 5 min. PCR clean-up was performed using AMPure XP beads (Beckman Coulter, Indianapolis, IN, USA) to purify the 16S V3 and V4 amplicon away from free primers and primer dimer species. Then, the next step was the index PCR, in this step attaches dual indices and Illumina sequencing adapters using the Nextera XT Index Kit (Illumina, San Diego, CA, USA), PCR conditions were: 95 °C for 3 min; 8 cycles of 95 °C for 30 s, 55 °C for 30 s, 72 °C for 30 s; 72 °C for 5 min, and hold at 4 °C. The pooled PCR products were purified using AMPure XP beads (Beckman Coulter, Indianapolis, IN, USA) before quantification. The resultant amplicons were sequenced at MiSeq (Illumina, USA), using paired-end (2x300nt) Illumina MiSeq sequencing system (Illumina, San Diego, CA, USA).

#### 2.4.3. Taxonomic Analysis

Galaxy [47] with the Mothur tool was used for quality assessment of raw reads, which were assembled and screened based on the minimum length of 250 bp and a maximum length of 550 bp. MG-RAST (metagenomics analysis server) [48] using the Ribosomal Database Project (RDP) for analyses of all sequences. The input processing steps in MG-RAST included demultiplexing, quality filtering, length filtering, dereplication, and removal of model organism sequences. The preprocessing options and details of data provided were: FASTQ sequences were filtered using a dynamic trimming. Fifteen was the specific lowest Phred quality score that was counted as a high-quality base and the sequences containing five bases below the value score 15 were trimmed. The raw microbiologic data were reported as relative abundances at the taxonomic levels of phylum, family, and genus. Microbial community diversity, including alpha diversity was analyzed. The observed relative abundance of each taxon was estimated by counting the number of reads for each taxon and then normalizing by the total number of reads per sample.

### 2.5. Statistical Analysis

Data are given as the mean ± standard mean error or median and range. *P*-values were obtained from the Mann–Whitney U-test or ANOVA test, as appropriate. *P* value < 0.05 value was considered to be statistically significant. Labeled means or medians without a common letter differ, *P* < 0.05. *P*-values were obtained from H Kruskal–Wallis tests corrected by Bonferroni post-hoc test when AMR and ANMR groups were compared with the control group. Principal component analysis (PCA) was used to maximize the information gained for the predominant variables from diet. This mathematical model calculates new variables (principal components) that account for the variability in the metagenomic data and enables the study of covariances or correlations between variables (e.g., total carbohydrates, total fat, among others). The combination of diet variables with the greatest amount of variability is the first principal component. The subsequent components (second and third principal components) describe the maximum amount of remaining variability [49,50]. All of the analyses were performed using the statistical package SPSS (SPSS Inc., Chicago, IL, USA). Boxplots were generated using the R software utilizing the ggplot2 package.

## 3. Results

Table 1 shows the general characteristics of ASD and healthy children participants.

Table 1 also shows the results of the Battelle, Childhood Autism Rating Scale (CARS) and Pervasive Developmental Disorder Behavior Inventory (PDDBI) psychological tests for both, children with AMR and ANMR, which were significantly different between each of them. Regardless of that, only two ASD children out of 48, reported frequent abdominal pain but no other gastrointestinal symptoms.

### ASD Children Show Fecal Metagenomic Differences Compared to Healthy Children

Table 2 shows the relative abundances of predominant phyla and classes between healthy children and children with ASD. All data are classified according to mental regression.

Following quality control, an average of 96,417 (range 25,633–263,373), 229,679 (12,644–587,681) and 223,197 (range 15,611–641,292) reads per sample were obtained for healthy children, children with ANMR, and children with AMR, respectively. Reads were classified into more than 4000 different taxons. All samples were rarefied to prevent bias due to sampling depth. At phylum level, *Actinobacteria* and *Proteobacteria* were higher in children with ASD compared to healthy group. For the mental regression, ANMR group showed augmented levels in *Actinobacteria* phylum and class compared with the healthy group, whereas children with AMR reported augmented *Proteobacteria* relative abundances compared with the control and ANMR groups. At class level, we found the same differences for *Actinobacteria* class and higher levels for *Bacilli*, *Erysipelotrichi*, and *Gammaproteobacteria* in the comparison of children with ASD and healthy group. Alpha diversity was similar between groups.

**Figure 1:**
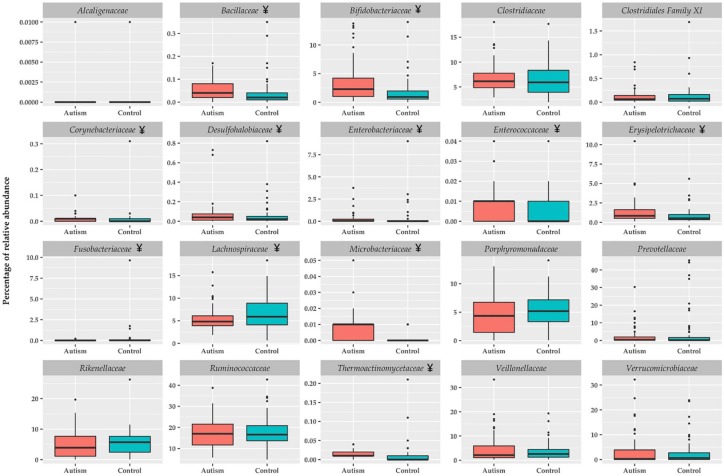
Relative abundance of family bacterial taxon in feces of children with ASD and healthy children. Boxplots were generated using the R software utilizing the ggplot2 package, and represent the most important bacterial families ^¥^ *P* < 0.05 vs. control group.

**Figure 2:**
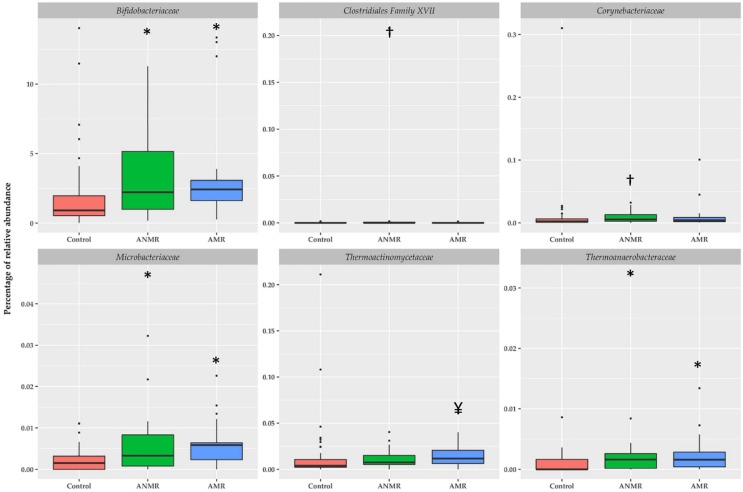
Relative abundances of selected bacteria according to family taxon in feces of children with ASD classified by mental regression and healthy children. Boxplots were generated using the R software utilizing the ggplot2 package and represent bacterial families * *P* < 0.05 vs. control group, ^†^ *P* < 0.05 vs. control and AMR groups, and ^¥^ *P* < 0.05 vs. control and ANMR groups using H Kruskal-Wallis test corrected by Bonferroni post-hoc test.

Figure 1 shows the bacterial families with higher representation in the intestinal microbiota. Bacillaceae, Bifidobacteriaceae, Corynebacteriaceae, Desulfohalobiaceae, Enterobacteriaceae, Enterococcaceae, Erysipelotrichaceae, Fusobacteriaceae, Microbacteriaceae, and Thermoactinomycetaceae were significantly higher in children with ASD compared to the healthy group. Only Lachnospiraceae showed to be lower in children with ASD in comparison to the healthy group. At family level, the differences between children with ASD and the healthy group were more notorious in relative abundances with values less than 0.1%. Figure 2 shows the specific differences among children with ANMR, AMR and the healthy group. Children with ANMR and AMR had higher levels of Bifidobacteriaceae, Microbacteriaceae, and Thermoanaerobacteraceae compared to the healthy group. Corynebacteriaceae and Clostridiales Family XVII were exclusively higher in children with ANMR, whereas Thermoactinomycetaceae family was significantly increased in children with AMR.

At genus and species levels, *Bacillus*, *Bifidobacterium*, *Butyrivibrio*, *Enterococcus*, *Hespellia*, *Prevotella*, *Clostridium bolteae*, and *Clostridium difficile* were significantly higher in children with ASD compared to healthy group. Children with AMR and ANMR had higher levels of *Bifidobacterium* and *Clostridium bolteae* compared to the healthy group, whereas only *Enterococcus* was significantly higher in children with AMR (Table 3).

Changes in intestinal microbiota might be related to food patterns and nutrient intakes. Hence, principal component analyses (PCA) were performed with bacterial and food consumption variables. In healthy children, component one was defined for fish, red meat and processed cold meat variables, those that were related inversely with cereal and milk consumption. Bacterial variables in component one were *Clostridium difficile* and *Butyrivibrio*, which correlated inversely with *Prevotella*. Children with ASD showed different nutritional variables related to component one, cakes and pastry with red and processed cold meat, as well as fish consumption were associated negatively with vegetables consumption. Finally, *Hespellia* was the only bacterial variable reported in component one.

Concerning children with AMR, we observed that variables in component one were similar to children with ASD, whereas children with ANMR had dissimilar variables, such as cereal and eggs and white meat consumption, and *Enterococcus*, *Bacillus* and *Butyrivibrio*.

Component two in healthy children included bacterial variables related in a positive way; we found here *Bifidobacterium* and *Enterococcus*. Food variables were snacks, cakes and pastry consumption, which correlated negatively with eggs and white meat. Finally, the component three was demarcated only through bacterial variables; here *Hespellia*, *Clostridium bolteae*, and *Bacillus* are presented. Children with ASD had a component two with *Enterococcus* and *Bacillus* that correlated inversely with *Clostridium difficile*, food variables were cereal, eggs and white meat, and milk products. Component three in children with ASD had a mix between bacterial variables and snack and soft drink consumption, here *Bifidobacterium*, *Clostridium bolteae*, and snack and soft drink consumption were correlated negatively with *Butyrivibrio* and *Prevotella*.

Component two in children with ANMR was defined by *Clostridium difficile*, *Hespellia* and fish consumption correlated negatively with vegetables and milk products consumption, and, component three was exclusively determined by bacterial variables, here *Bifidobacterium* and *Clostridium bolteae* were related negatively with *Prevotella*.

**Figure 3:**
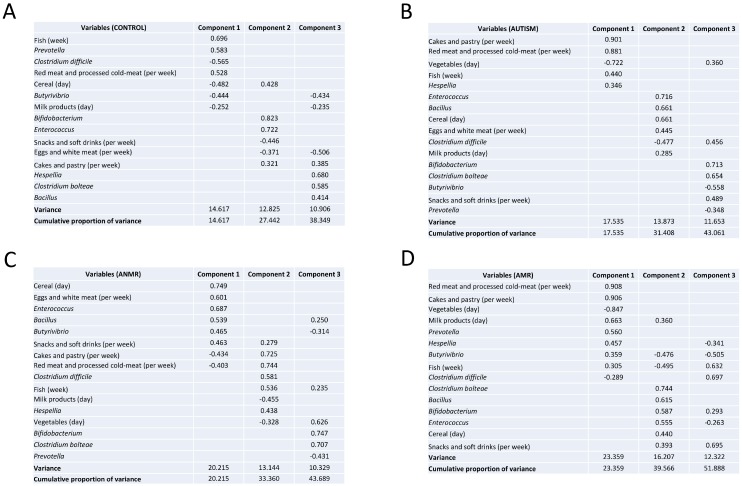
Principal component analysis between intestinal microbiota and food consumption in children with ASD and healthy children. Extraction of the initial set of uncorrelated components was accomplished with the principal factor method and then the orthogonal rotation of components was used to facilitate interpretation. Factor loading was used to interpret the factor structure. Loadings are equivalent to Pearson correlation coefficients, and a higher loading indicates a stronger relation between a factor and an observed variable. Strong loading was defined as a value ≥ 0.6; moderate as 0.4–0.59 and low as <0.4. (**A**) Control group, (**B**) Children with ASD, (**C**) Children with ANMR, (**D**) Children with AMR. ASD, children with autism spectrum disorders; AMR, children with autism mental regression; ANMR, children with autism non-mental regression.

Finally, component two in children with AMR was defined through bacterial variables; *Clostridium bolteae*, *Bacillus*, *Bifidobacterium* and *Enterococcus* correlated positively with cereal and snacks and soft drinks consumption (Figure 3).

## 4. Discussion

The present study analyzes the fecal microbiota in children with ASD, classified according to mental regression phenotypes using a metagenomic approach. The major findings were that fecal samples from children with ASD exhibited several differences compared to a healthy group, especially in *Actinobacteria* and *Proteobacteria*, as these two phyla were significantly higher in children with ASD. In addition, *Actinobacteria* class, *Bacilli*, *Erysipelotrichi*, and *Gammaproteobacteria* levels were increased in children with ASD. Moreover, Bacillaceae, Bifidobacteriaceae, Corynebacteriaceae, Desulfohalobiaceae, Enterobacteriaceae, Enterococcaceae, Erysipelotrichaceae, Fusobacteriaceae, Microbacteriaceae, and Thermoactinomycetaceae, and *Bacillus*, *Bifidobacterium*, *Butyrivibrio*, *Enterococcus*, *Hespellia*, *Prevotella*, *Clostridium bolteae*, and *Clostridium difficile* were significantly augmented in children with ASD. When children with ASD were divided according MR, *Corynebacteriaceae* and *Clostridiales* Family XVII were solely higher in children with ANMR, whereas *Proteobacteria*, *Thermoactinomycetaceae* and *Enterococcus* abundances were exclusively higher in children with AMR. These findings support previous studies reporting changes in intestinal microbiota from children with ASD, and add new information regarding mental regression, a new variable in ASD that could influence the intestinal microbiota profile.

Patients with ASD who present gastrointestinal symptoms might display significant behavioral manifestations, such as anxiety, self-injury and aggression [11]. We mentioned earlier that gut microorganisms can affect brain functions through the vagus nerve [31], and accumulating evidence demonstrates that this gut microbiota is directly or indirectly associated with ASD symptoms, in part by influencing the immune system and metabolism [51,52]. Changes in the composition of gut microorganisms can affect both the enteric nervous system and the central nervous system (CNS), thereby indicating the existence of a microbiota—gut—brain axis [31].

*Actinobacteria* is an important phylum exhibiting differences between children with ASD compared with healthy ones. Four specific studies [18,21,27,53] have reported decreased *Actinobacteria* in children with ASD, whereas a study in 2005 showed no differences [54]. Opposite to that reported in previous work, our study showed an increase in that phylum. According to mental regression, children with ANMR had significantly higher *Actinobacteria* in comparison with the healthy group. *Bifidobacterium* represents a genus within the *Actinobacteria* phylum specially found in infants during lactation. Bifidobacteria are early colonizers of gut microbiota, and have reported properties in the metabolism of dietary components, and even effects over maturation of the immune system [55,56]. In addition, consumption of yogurt and other fermented dairy products is associated with an increase of fecal bifidobacteria [57,58,59]. Children with ASD, ANMR and AMR had significantly higher amounts of *Bifidobacterium* in comparison to the healthy group, which may be related to a high consumption of dairy products.

In *Akkermansia* genus, we found *A. muciniphila* an important bacterium able to prevent the development of obesity in animal models. A pasteurized version of *A. muciniphila* was capable to reduce fat mass development, insulin resistance, and dyslipidemia in mice; moreover, the pasteurized bacterium modulated both the host urinary metabolome and intestinal energy absorption [60]. *Akkermansia* genus was not different in none comparison in our studied children. In fact, *Akkermansia* genus was similar in all groups, showing a wide variation between the evaluated samples. The results in this genus are contradictory; Kang et al. [61] found similar results with our study, unchanged genus in the comparison with children with ASD. De Angelis et al. [53] reported increased amount in *Akkermansia*, and in contrast, Wang et al. [18] found the opposite.

*Prevotella* is a large genus that includes almost 40 different species with a vast genomic diversity. It has been suggested that *Prevotella* is a beneficial bacteria as it is associated with a plant-rich diet, however it is also linked to chronic inflammatory conditions, such as arthritis and mucosal and systemic T-cell activation in untreated human immunodeficiency virus type 1 (HIV-1) infection [62,63]. The case of *Prevotella* is similar to the previous genus *Akkermansia*, unchanged relative abundance in Wang study [18], decreased levels in Kang et al. [61] study and finally, augmented levels in De Angelis et al. [53] study. For *Prevotella*, we report a significant increase in children with ASD.

Changes in the gut microbiota associated with ASD might be related to an increased permeability of the intestinal tract of individuals, referred to as a “leaky gut” [64], but this factor is related directly with other factors, such as, immune response. Previous studies have demonstrated that ASD animal models present defects in the gastrointestinal barrier, resulting in the entry of the toxins and bacterial products into the bloodstream, which influence brain function [14,65]. These arguments were supported with the idea of level changes in some immune cells and cytokines, such as, interleukin (IL)-6, IL-1β, IFN-γ, and TNF-α, among others. However, plasma cytokine levels in our children with ASD compared to the levels in the healthy control group, only showed differences in the nerve growth factor, but the aforementioned cytokines were not significantly different [22]. Although microbiota changes are related with immunological responses, profound differences require more variables to explain the present variance. Accordingly, Lovene et al. found that in few ASD-positive samples, with low-mild augmented calprotectin values, no correlation was found with either increase or decrease of bacterial/yeast species. A correlation was, however, present with disease degree (CARS score), thus possibly confirming that gut inflammation also could participate to gut discomfort ending in influence on behavior [24].

An association between high levels of *Sutterella* species and gastrointestinal disturbances in children with ASD was reported. These findings in ileal and cecal biopsy samples have demonstrated that *Sutterella*, a bacterium with a low abundance in the microbiota, is a major component of the microbiota in over half of children with ASD and gastrointestinal dysfunction and is absent in healthy children with only gastrointestinal dysfunction [66]. Microbes are different from the places where they are extracted [67], in our study *Sutterella* was not detected in none analyzed group. This might be related to the usual Mediterranean diet consumed in Spain compared to other countries.

Maternal obesity during pregnancy and gestational diabetes alter the gut microbiota and might be associated with ASD in humans [68]. As shown by Buffington et al., a maternal high-fat diet induces dysbiosis and autism-like phenotypes in mice, and *Lactobacillus reuteri* restores these alternations [69]. Diet might have a tremendous influence in the development of ASD. However, a systematic review concluded that the evidence to support a gluten-free, casein-free diet is limited and weak, considering that dietary restrictions might be responsible for further social withdrawal and integration, in addition to potential adverse clinical effects [70]. Elimination diets for ASD patients should only be initiated after reaching a diagnosis of an adverse food reaction. On the other hand, the administration of probiotics can be useful for restoring the microbial balance in the intestine and ameliorating gastrointestinal symptoms. Some evidence has accumulated regarding the possible role of probiotics in modulating some neurological symptoms [71]. Because ASD patients presented GI dysbiosis [20,21], which may exacerbate the disease [19]. However, in our study, alpha diversity was not significantly different in all tested groups.

We have included in our study, a dietary dimension, transforming the consumed portions with the recommendations of Spanish Society of Community Nutrition (SENC) Food Guide Pyramid in scholar age to determine or assess potential relationships between microbiota and diet [45]. The profile in healthy children was determined by consumption of fish, red meat and processed cold meat, cereal and milk products, whereas children with ASD have cakes and pastry with red meat and processed cold meat, and fish consumption, which related negatively with vegetables consumption. Moreover, bacterial variables in component one, were *Clostridium difficile* and *Butiryvibrio*, which correlated inversely with *Prevotella* in healthy children. In contrast, in children with ASD *Hespellia* was the only bacterial variable reported in component one. According to mental regression, children with ANMR seem to exhibit a microbiota profile typical of children with ASD than AMR group; however further analyses are needed to confirm these results.

A limitation of the present study according to microbiota was the biological material analyzed; we just were able to evaluate fecal samples, and others, such as, buccal samples, urine samples or saliva will be required to connect these findings with an overall “microbiome” view. To better understand the role of the microbiota and the interplay between nutrition and the microbiota in ASD, future studies should systematically investigate the role of food and nutrition in the microbial composition of children with ASD and stress the importance of analyzing the microbiota in the context of diet and medication [72]. In addition, multicomponent analyses including microbiota patterns, genetic and metabolomics approaches, and nutrition assessment are required to understand how children with ASD can exhibit different phenotypes and behaviors.

## 5. Conclusions

In conclusion, several differences between children with ASD compared with healthy group were detected; *Actinobacteria* and *Proteobacteria* at phylum level, and *Actinobacteria*, *Bacilli*, *Erysipelotrichi*, and *Gammaproteobacteria* at class level were higher in children with ASD. Additionally, *Proteobacteria* levels were augmented exclusively in children with AMR. Mixed bacterial and nutritional variables showed differential patterns in children with ASD, ANMR and AMR. Furthermore, Corynebacteriaceae and *Clostridiales Family* XVII were solely higher in children with ANMR, whereas *Proteobacteria*, Thermoactinomycetaceae and *Enterococcus* abundances were exclusively higher in children with AMR. Further analyses mixing omics technologies, diet questionnaires and inflammation pathways are needed in larger ASD cohorts to confirm the results of the present study in terms of the population of children in Spain.

## Figures and Tables

**Table 1:** Descriptive characteristic of autism spectrum disorder and healthy children participants.

Variables	ASD			Control (*n* = 57)	*P* Value
	ANMR (*n* = 30)	AMR (*n* = 18)	Total (*n* = 48)		
Age (months)	44.51 ± 2.06	43.69 ± 2.7	44.19 ± 1.6	51.00 ± 2.59	n.s.
Weight (kg)	16.57 ± 0.56	17.10 ± 0.95	16.77 ± 0.50	17.1 ± 0.6	n.s.
Height (cm)	103.3 ± 1.55	101.50 ± 1.92	102.5 ± 1.20	102.0 ± 1.5	n.s.
BMI (kg/cm^2^)	15.52 ± 0.33	16.37 ± 0.39	15.86 ± 0.26	16.2 ± 0.2	n.s.
Battelle test	59.6 ± 2.6	47.5 ± 2.6	54.98 ± 2.0	-	0.003
CARS test	30.6 ± 1.1	35.9 ± 1.9	32.7 ± 1.1	-	0.021
PDDBI test	46.8 ± 2.3	53.6 ± 1.6	50.9 ± 1.6	-	0.026

Data are given as the mean ± standard error of the mean. *P*-values were obtained from the Mann-Whitney U-test, or ANOVA test, as appropriate. *P* < 0.05 value was considered to be statistically significant. Abbreviations: ASD, children with autism spectrum disorders; AMR, children with autism mental regression; ANMR, children with autism non-mental regression; BMI, body mass index; CARS, Childhood Autism Rating Scale test; n.s., non-significant; PDDBI, Pervasive Developmental Disorder Behavior Inventory.

**Table 2:** Relative abundances of bacteria in fecal microbiota of children with ASD and healthy children.

Variables	ASD			
	ANMR Group (*n* = 30)	AMR Group (*n* = 18)	Total ASD (*n* = 48)	Healthy Control Group (*n* = 57)
*Actinobacteria* (phylum)	2.6 (0.6–14.9) ^a^	3.2 (0.3–16.8) ^ab^	2.9 (0.3–16.8) *	1.8 (0.1–18.3) ^b^
*Bacteroidetes* (phylum)	43.4 (2.0–58.1)	39.5 (11.0–51.7)	43.0 (2.0–58.1)	42.9 (8.5–67.9)
*Firmicutes* (phylum)	45.4 (31.0–82.9)	44.0 (19.6–61.6)	44.7 (19.6–82.9)	42.2 (19.2–81.1)
*Proteobacteria* (phylum)	0.2 (0.0–4.1) ^a^	0.4 (0.1–2.8) ^b^	0.4 (0.0–4.1) *	0.2 (0.0–8.9) ^a^
*Verrucomicrobia* (phylum)	0.1 (0.0–24.5)	1.1 (0.0–30.9)	0.3 (0.0–30.9)	0.7 (0.0–23.2)
*Actinobacteria* (class)	2.6 (0.6–14.9) ^a^	3.2 (0.3–16.8) ^ab^	2.9 (0.3–16.8) *	1.8 (0.0–18.3) ^b^
*Bacilli* (class)	0.4 (0.1–6.4)	0.4 (0.0–1.9)	0.4 (0.0–6.4) *	0.3 (0.0–2.4)
*Bacteroidia* (class)	43.3 (1.9–58.1)	39.5 (7.7–51.7)	42.9 (1.9–58.1)	42.9 (8.5–67.9)
*Clostridia* (class)	36.0 (22.9–51.3)	35.6 (17.2–57.1)	35.6 (17.2–57.1)	37.5 (12.5–65.8)
*Deltaproteobacteria* (class)	0.1 (0.0–0.8)	0.1 (0.0–1.4)	0.1 (0.0–1.4)	0.07 (0.0–0.9)
*Erysipelotrichi* (class)	0.6 (0.2–10.1)	0.9 (0.1–4.8)	0.8 (0.1–10.1) *	0.5 (0.2–5.5)
*Gammaproteobacteria* (class)	0.1 (0.0–3.7)	0.2 (0.0–2.7)	0.1 (0.0–3.7) *	0.04 (0.0–8.8)
*Negativicutes* (class)	4.6 (0.2–32.4)	1.9 (0.4–15.4)	2.8 (0.2–32.4)	2.9 (0.5–20.8)
*Verrucomicrobiae* (class)	0.1 (0.0–24.5)	1.1 (0.0–30.9)	0.3 (0.0–30.9)	0.7 (0.0–23.2)
Unclassified sequences derived from Bacteria	4.7 (1.4–13.3) ^a^	9.7 (2.2–32.3) ^b^	5.9 (1.4–32.3)	8.0 (1.4–31.8) ^b^
Alpha diversity	33.5 (17.0–86.0)	28.5 (9.0–55.0)	30.5 (9.0–86.0)	32.0 (12.0–62.0)

Data are given as median and range. * *P*-values were obtained from the Mann-Whitney U-test. Labeled medians with identical letters are not significant. Different letters means significant differences (*P* < 0.05) and were calculated using a H Kruskal–Wallis test corrected by Bonferroni post-hoc test. ASD, children with autism spectrum disorders; AMR, children with autism mental regression; ANMR, children with autism non-mental regression. Table 2 shows only the phylum and class abundances with a value higher than 0.1%.

**Table 3:** Relative abundances of fecal bacteria (genus and species) in children with ASD classified by mental regression and healthy children.

Variables	ASD			Control (*n* = 57)
	ANMR (*n* = 30)	AMR (*n* = 18)	Total (*n* = 48)	
*Akkermansia*	0.06 (0.0–24.8)	1.1 (0.0–32.3)	0.30 (0.0–32.3)	0.7 (0.0–24.2)
*Alistipes*	3.9 (0.0–13.8)	3.8 (0.0–20.0)	3.9 (0.0–20.0)	5.7 (0.0–26.1)
*Bacillus*	0.03 (0.0–0.1)	0.03 (0.0–0.1)	0.03 (0.0–0.1) *	0.02 (0.0–0.4)
*Bacteroides*	30.3 (1.1–60.8)	23.2 (4.0–48.4)	28.6 (1.1–60.8)	29.4 (2.4–51.3)
*Bifidobacterium*	2.2 (0.2–13.8) ^a^	2.4 (0.3–14.2) ^a^	2.3 (0.2–14.2) *	0.9 (0.0–14.0) ^b^
*Butyrivibrio*	1.0 (0.2–5.3)	1.0 (0.1–4.7)	1.0 (0.1–5.3) *	1.4 (0.2–6.8)
*Clostridium*	6.1 (3.0–11.7)	5.7 (3.0–16.9)	5.8 (3.0–16.9)	5.4 (1.8–16.9)
*Collinsella*	0.4 (0.0–4.4)	0.2 (0.0–8.2)	0.4 (0.0–8.2)	0.5 (0.0–5.0)
*Desulfovibrio*	0.001 (0.0–0.5)	0.002 (0.0–1.3)	0.002 (0.0–1.3)	0.0005 (0.0–0.3)
*Enterococcus*	0.002 (0.0–0.04) ^a^	0.004 (0.0–0.01) ^b^	0.004 (0.0–0.04) *	0.001 (0.0–0.04) ^a^
*Eubacterium*	4.0 (0.5–10.9)	2.6 (0.3–8.4)	3.6 (0.3–10.9)	2.6 (0.5–13.5)
*Faecalibacterium*	11.7 (1.4–22.8)	9.6 (1.8–37.0)	10.7 (1.4–37.0)	11.4 (2.7–37.1)
*Hespellia*	0.2 (0.0–1.9)	0.2 (0.0–0.7)	0.2 (0.0–1.9) *	0.1 (0.0–0.9)
*Lactobacillus*	0.05 (0.0–3.5)	0.03 (0.0–0.6)	0.04 (0.0–3.5)	0.03 (0.0–1.0)
*Parabacteroides*	2.6 (0.0–8.3)	2.1 (0.0–5.5)	2.4 (0.0–8.3)	1.9 (0.0–10.0)
*Prevotella*	0.3 (0.0–30.2)	0.2 (0.0–11.9)	0.3 (0.0–30.2) *	0.1 (0.0–43.7)
*Ruminococcus*	3.1 (1.1–10.3)	3.3 (0.9–15.8)	3.1 (0.9–15.8)	3.2 (0.7–26.5)
*Veillonella*	0.4 (0.1–34.9)	0.6 (0.0–5.8)	0.5 (0.0–34.9)	0.7 (0.0–19.3)
*Bacteroides fragilis*	0.33 (0.0–9.1)	0.39 (0.0–7.9)	0.35 (0.0–9.1)	0.34 (0.0–32.1)
*Bacteroides vulgatus*	2.9 (0.0–28.1)	1.2 (0.0–36.7)	1.4 (0.0–36.7)	8.5 (0.0–36.3)
*Clostridium bolteae*	0.1 (0.0–1.7) ^a^	0.1 (0.0–1.2) ^a^	0.1 (0.0–1.7) *	0.04 (0.0–1.4) ^b^
*Clostridium difficile*	0.08 (0.0–0.6)	0.04 (0.0–0.6)	0.09 (0.0–0.6) *	0.06 (0.0–0.8)
*Faecalibacterium prausnitzii*	11.3 (1.4–21.9)	9.1 (1.7–36.0)	10.3 (1.4–36.0)	11.3 (2.6–35.7)
*Ruminococcus gnavus*	0.3 (0.0–4.5)	0.2 (0.0–1.7)	0.3 (0.0–4.5)	0.3 (0.0–7.6)
*Ruminococcus torques*	0.1 (0.0–0.9)	0.1 (0.0–3.2)	0.1 (0.0–3.2)	0.08 (0.0–5.0)

Data are given as the median and range. * *P*-values were obtained from the Mann-Whitney U-test. Labeled medians with identical letters are not significant. Different letters means significant differences (*P* < 0.05) and were calculated using a H Kruskal Wallis test corrected by Bonferroni post-hoc test. ASD, children with autism spectrum disorders; AMR, children with autism mental regression; ANMR, children with autism non-mental regression. Table three shows only the important genus and species abundances.
